# Prevalence of Neurocysticercosis in People with Epilepsy in the Eastern Province of Zambia

**DOI:** 10.1371/journal.pntd.0003972

**Published:** 2015-08-18

**Authors:** Kabemba E. Mwape, Joachim Blocher, Jasmin Wiefek, Kathie Schmidt, Pierre Dorny, Nicolas Praet, Clarance Chiluba, Holger Schmidt, Isaac K. Phiri, Andrea S. Winkler, Sarah Gabriël

**Affiliations:** 1 Department of Clinical Studies, School of Veterinary Medicine, University of Zambia, Lusaka, Zambia; 2 Department of Neurology, Medical University Center Göttingen, Göttingen, Germany; 3 Department of Neurology, Elbe-Kliniken Stade/Buxtehude GmbH, Stade, Germany; 4 Europe-University of Flensburg, Flensburg, Germany; 5 Department of Neuroradiology, Medical University Center Göttingen, Göttingen, Germany; 6 Department of Biomedical Sciences, Institute of Tropical Medicine, Antwerp, Belgium; 7 Levy Mwanawasa General Hospital, Lusaka, Zambia; 8 Department of Neurology, Technical University Munich, Munich, Germany; Universidad Nacional Autónoma de México, MEXICO

## Abstract

Zambia is endemic for *Taenia solium* taeniosis and cysticercosis. In this single-centered, cross-sectional, community-based study, the role of neurocysticercosis (NCC) as a cause of epilepsy was examined. People with epilepsy (PWE, n = 56) were identified in an endemic area using a screening questionnaire followed by in-depth interviews and neurological examination. Computed tomography (CT) was performed on 49 people with active epilepsy (PWAE) and their sera (specific antibody and antigen detection, n = 56) and stools (copro-antigen detection, n = 54) were analyzed. The CT scan findings were compared to a group of 40 CT scan controls. Of the PWE, 39.3% and 23.2% were positive for cysticercal antibodies and antigens, respectively, and 14.8% for coproantigens (taeniosis). Lesions highly suggestive of NCC were detected in 24.5% and definite NCC lesions in 4.1% of CT scans of PWAE. This compares to 2.5% and 0%, respectively, in the control CT scans. Using the Del Brutto diagnostic criteria, 51.8% of the PWAE were diagnosed with probable or definitive NCC and this rose to 57.1% when the adapted criteria, as proposed by Gabriël et al. (adding the sero-antigen ELISA test as a major criterion), were used. There was no statistically significant relationship between NCC, current age, age at first seizure and gender. This study suggests that NCC is the single most important cause of epilepsy in the study area. Additional large-scale studies, combining a community based prevalence study for epilepsy with neuroimaging and serological analysis in different areas are needed to estimate the true impact of neurocysticercosis in endemic regions and efforts should be instituted to the control of *T*. *solium*.

## Author Summary

Neurocysticercosis, an infection caused by the larval stage of *Taenia solium* (the pork tapeworm), is a preventable and treatable disease and one of the main causes of epilepsy in low-income countries. Studies in Zambia have indicated high endemicity of *T*. *solium* in rural communities where a high prevalence of epilepsy has been reported. It was therefore important to analyze the role of neurocysticercosis as a cause of epilepsy. Diagnosis of the condition, however, relies on neuroimaging techniques, which are not routinely available in our study area. In this imaging-based study, people with epilepsy were identified, examined by a neurologist, their brains scanned using computed tomography, and their stools and sera analyzed for specific antigens and antibodies. The study suggests that 57.1% of these people suffered from neurocysticercosis, making it the single most important cause of epilepsy in the study area. The results show the need to institute control measures to alleviate the suffering of the affected communities. In summary, the recognition of neurocysticercosis as an important cause of epilepsy will aid in the treatment and prevention of epilepsy in affected communities and alleviate the burden of neurocysticercosis on the rural people.

## Introduction

Neurocysticercosis (NCC) is a parasitic infection of the brain caused by the metacestode larval stage of the pork tapeworm *Taenia solium* and occurs primarily in low-income countries. Pigs are the natural intermediate host for the tapeworm, while humans are the only definitive host but may accidentally also serve as the intermediate host [1]. Humans become tapeworm carriers (taeniosis) after ingestion of undercooked pork that is infected with cysticerci (cysticercosis). Humans and pigs acquire cysticercosis after ingestion of infective eggs that are passed in stool by a tapeworm carrier. The cysts have the propensity to localize in the brain leading to NCC [1]. NCC can cause a wide range of neurological disorders including epileptic seizures, headaches and focal neurological symptoms/signs [2–4]. It is reported as one of the major causes of acquired epilepsy in endemic areas [5, 6], which has profound social, physical and psychological consequences. Population based studies have reported that approximately 30% of cases of epileptic seizures are attributable to NCC [7, 8].

The prevalence of epilepsy as determined in community-based studies throughout Africa shows varying results depending on the study population and the methodologies used, ranging from 5.2–74.4/1000 with a median of 15/1000 inhabitants [9, 10]. This is compared to high-income countries where the prevalence of active epilepsy is estimated to be about 4–8/1000 inhabitants [11]. Epilepsy represents the most common chronic neurological disorder in sub-Saharan Africa, and involves unprovoked recurrent (two or more) afebrile seizures that may result in injury, disability and social stigmatization [12, 13]. For individuals with epilepsy in many low-income countries, including Zambia, the etiology of the disease often remains unclear.

The endemicity of *T*. *solium* infections in Zambia has been mentioned in many reports. Porcine cysticercosis prevalence estimates have been reported to range from 8.2–64.2% [14] while human cysticercosis is reported to range from about 6–13% (based on circulating antigen detection) in studies carried out in the Eastern province [15, 16]. The Eastern province harbors almost 50% of the total number of pigs in the country, which are mostly reared under the small-scale management system [17]. Due to the lack of sanitary facilities such as latrines, free-ranging pigs have access to human feces. Such conditions increase the risk of both porcine cysticercosis and human taeniosis/cysticercosis infection. The high percentage of active cysticercosis infections (12.5%), determined in a study in Katete district (Eastern province) [15], indicates the urgent need to identify the importance of NCC as a cause of epilepsy in this area. Therefore, the main objective of this cross-sectional study was to analyze the role of NCC as a cause of epilepsy in a study area of Katete district of the Eastern province of Zambia.

## Materials and Methods

### Ethical considerations

The University of Zambia Biomedical Research Ethics Committee (IRB0001131) and the Ethical Committee of the University of Antwerp, Belgium, both granted ethical clearance for the study (ITG:12084813). Further approval was sought from the Ministry of Health of Zambia, from the local district health authorities and the area chief. Meetings were held with the people in the villages through their leaders (headmen) to explain the purpose of the study, request their permission to conduct the study and also to invite them to participate. Further permission was sought from the individual subjects to enter the study after oral consent for the screening questionnaires and written informed consent for further examinations. The use of oral consent in the screening phase was explicitly approved by both ethics committees and no special documentation of the oral consent was required. For individuals below the consenting age, their parents were asked for informed consent on their behalf. All collected samples were assigned an identification number that was linked to the questionnaire, neurological examination, laboratory test results and neuroimaging results. For neuroimaging, participants that were diagnosed with active epilepsy were transported to Lusaka accompanied by two medical officials from the local rural health center. For children below the age of 16, a parent or guardian accompanied them to Lusaka. In collaboration with the regional health center Mtandaza all patients received free antiepileptic drugs according to national guidelines and niclosamide in case they were diagnosed with taeniosis. Patients with active NCC were offered a combination of dexamethasone and anthelmintic treatment in an inpatient setting and, according to the resolution of their symptoms a re-scan free of charge.

### Study site

**Fig 1:**
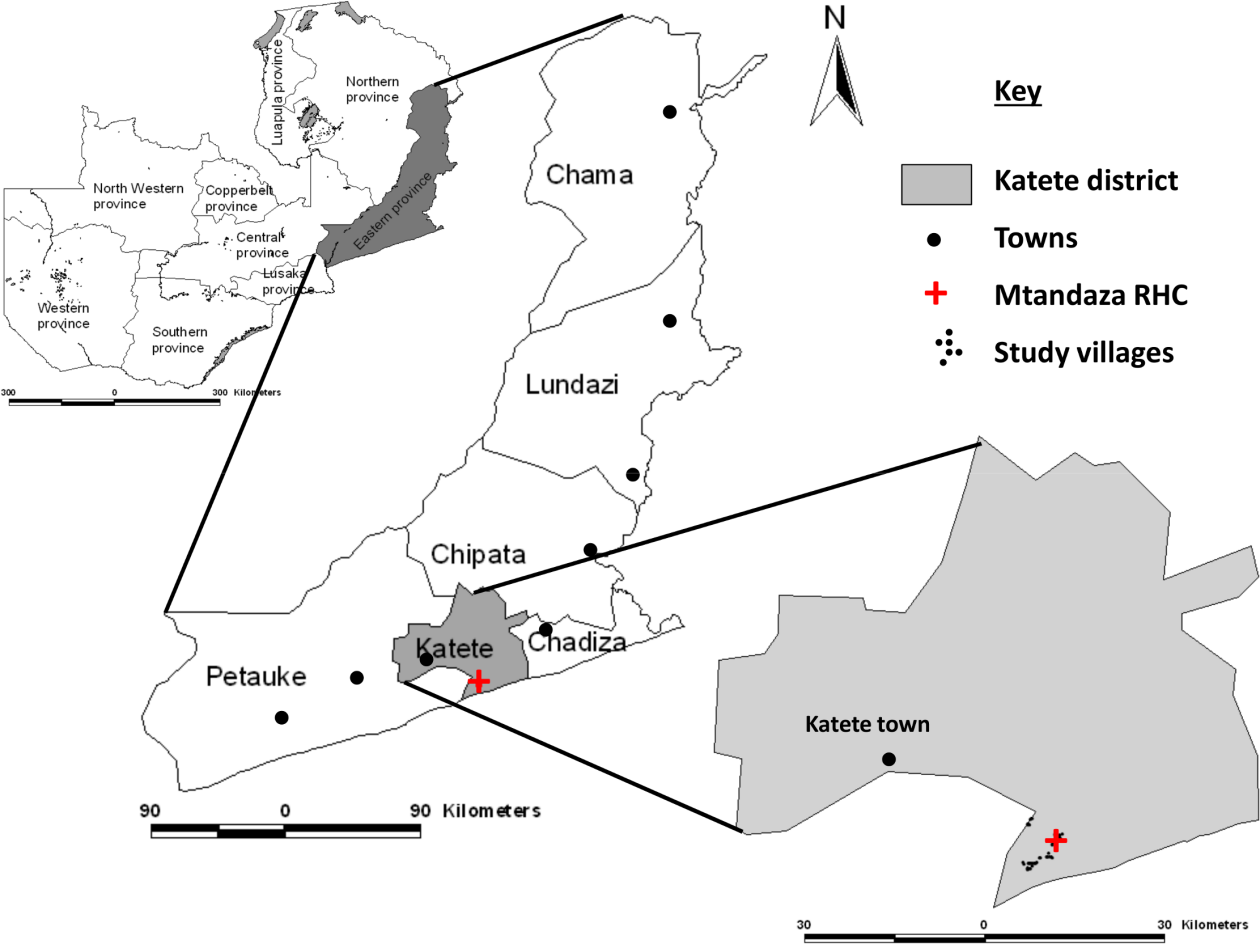
Map of Zambia showing the study area in Katete district of the Eastern province.

The study was carried out in Mtandaza community in Katete district of the Eastern province of Zambia (Fig 1). The Mtandaza Rural Health Center (RHC) provides health care in this community with a catchment population of approximately 20000. The people in this area practice subsistence agriculture raising animals like cattle, goats, pigs and chickens and growing crops like maize, groundnuts, bananas and cotton. Their homes are of adobe, have few sanitary facilities with hand pumps being the source of water for most villages. Pigs are commonly bought and sold among villagers and also to intermediary traders from nearby towns. Since sanitation is poor, pigs have access to the feces in nearby bushes that are used as latrines by the villagers.

The district of Katete was chosen because endemicity of human and porcine *T*. *solium* cysticercosis has been demonstrated during previous studies [16, 18]. The rural community was selected because of free-range pig keeping, absence of active ongoing sanitation programs, reports of people with epilepsy (PWE), and the common observation of cysticerci in slaughtered pigs. Furthermore, the RHC had shown willingness to collaborate with this study.

### Study design and recruitment of people with epilepsy

This was a single-center, cross-sectional and community-based study. In August 2012, the study began with the screening of all inhabitants of villages within a 7 km radius from the Mtandaza RHC. Local health workers, after undergoing basic questionnaire administration training, used a standardized screening questionnaire modified and adapted by Birbeck and Kalichi [19] to identify people with possible epilepsy (see S1 File). The screening questionnaire was administered door-to-door to any individual that lived in the study area and was willing to participate.

**Fig 2:**
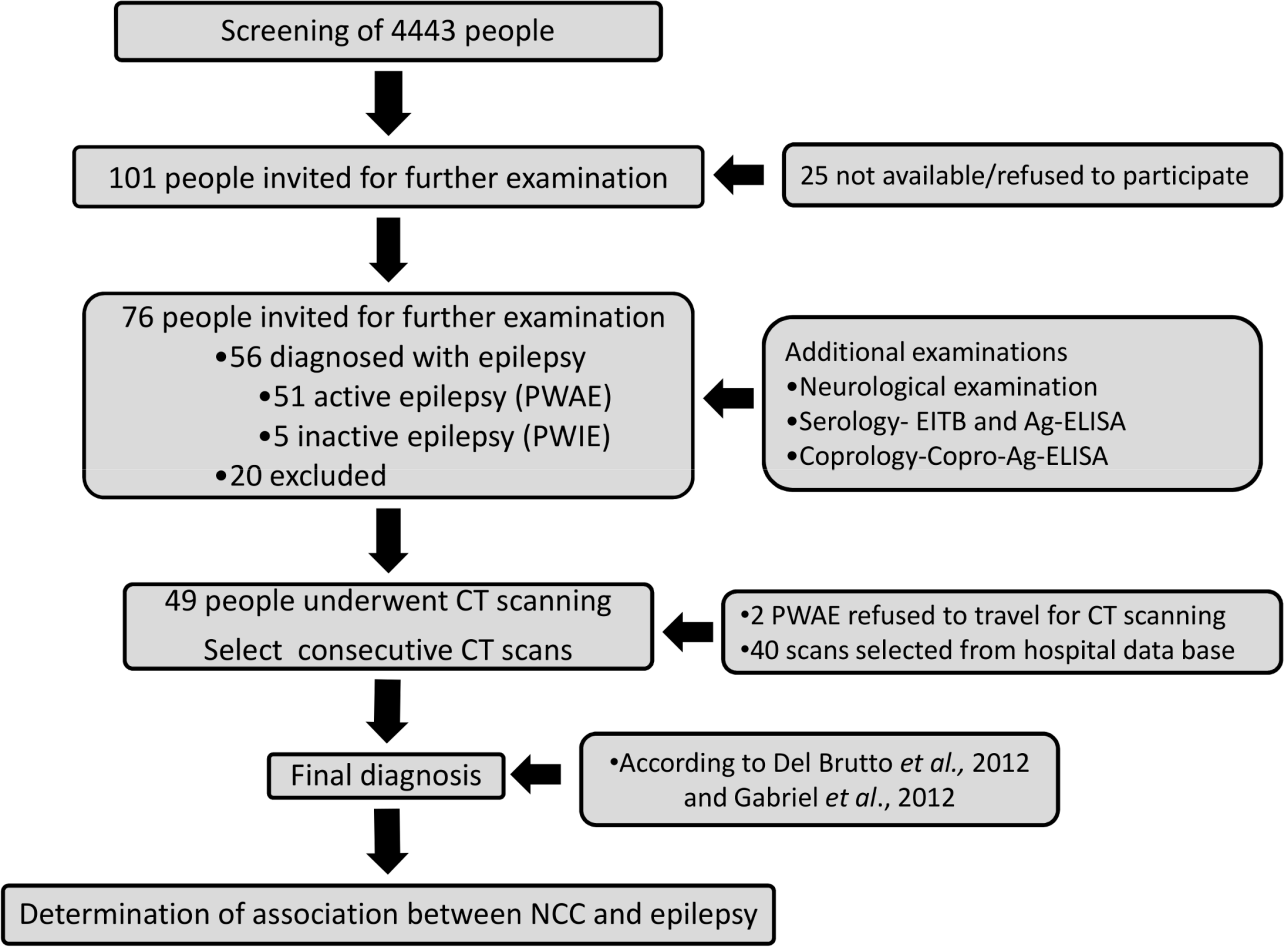
Draft of the study design.

Altogether, 4443 individuals were screened (Fig 2). Due to financial restriction the number of computed tomography (CT) scans had to be limited to 50. Therefore, the recruitment started with those people with the highest number of positive answers to the screening questions 1–8 and a negative answer in question nine. Finally, all people with a minimum of four positive answers and one with three positive answers, giving a total of 101 individuals, were invited for further examinations at the RHC. Twenty-five individuals were either not available for examination or refused to participate in the study. After written informed consent, we examined 76 individuals with suspected epilepsy. Twenty individuals dropped out because they had no epilepsy or they withdrew their consent (see also Table 1).

**Table 1:** Reasons for exclusion and number of individuals excluded from the study.

Reason for exclusion	Number
Only febrile seizures	6
No epileptic seizures	2
Alcohol provoked seizures	2
Other provoked seizures	2
Syncope	3
Single provoked seizure	2
Psychosis	1
Withdrawal of consent	2
**Total**	20

The 56 identified PWE provided a detailed medical history, underwent neurological examination and provided blood and stool samples for the immunological diagnosis of cysticercosis and taeniosis, respectively (Fig 2). Epilepsy was diagnosed according to a classification for resource poor settings, which is in agreement with the definitions of epilepsy by the International League against Epilepsy, but was adapted to local circumstances. The four major diagnostic groups were “Generalized seizures within specific age range”, “Generalized seizures outside specific age range”, “Generalized seizures with diffuse brain damage” and “Generalized seizures with focal signs”. The first two groups were distinguished by the age at onset of epilepsy being either within or outside the age span of six to 25 years [20]. Fifty-one patients were diagnosed with active epilepsy (PWAE), which was defined that the patient had had seizures within the last two years or had been on antiepileptic medication, while five had inactive epilepsy (PWIE) and did not fulfill the criteria mentioned before. The PWAE were transported 480 km to Lusaka and examined for lesions compatible with NCC by CT scanning of the brain at the public Levy Mwanawasa General Hospital.

### Sample collection and analyses

About 5 ml of blood were collected into sterile plain blood collecting tubes and allowed to clot. Serum was extracted from all the blood samples by first allowing them to stand at 4°C overnight and then centrifuging at 3000g for 15 minutes. The supernatant (serum) was then aliquoted into 1.8 ml vials and stored at -20°C until use. Submitted stool samples were placed in 10% formalin and stored until use. All the samples were analyzed in the Cysticercosis Working Group for Eastern and Southern Africa Reference Centre for Cysticercosis at the School of Veterinary Medicine at the University of Zambia in Lusaka.

The serum samples were tested for circulating cysticercal antigens using the monoclonal antibody based B158/B60 antigen enzyme linked immunosorbent assay (sero-Ag-ELISA) as described by Dorny et al. [21]. To determine the cutoff, the optical density (OD) of each serum sample was compared with a series of eight reference negative human serum samples at a probability level of p < 0.001 [21]. The serum samples were also tested for specific antibodies against cysticercosis using a commercial kit, Immunetics (Immunetics Inc., Boston, Massachusetts, USA). The assay was performed according to the manufacturer’s instructions. In brief, the assay is an enzyme linked immunoelectrotransfer blot (EITB), which uses seven purified *T*. *solium* antigens (diagnostic bands being Gp50, Gp42-39, Gp24, Gp21, Gp18, Gp14 and Gp13, whereby Gp stands for glycoprotein and the number is the molecular weight of each antigen expressed in kilodaltons). Reactions to any one or more of the bands are considered positive.

The stool samples were examined for copro-antigens using the polyclonal antibody based ELISA (copro-Ag-ELISA) as described by Allan et al. [22] with slight modifications [15]. To determine the test result, the OD of each stool sample was compared with the mean of a series of eight reference *Taenia* negative stool samples plus three standard deviations (cut-off).

### Performance of neuroimaging

Of the 51 PWAE, 49 (two refused) underwent CT examination at the Levy Mwanawasa General Hospital in Lusaka. The CT machine used was a Neusoft helical multi-slice scanner (Neusoft Medical Systems, Shenyang, China). The thickness of slices for the whole scan was 5 mm. Intravenous contrast medium was not applied. A set of 101 consecutive cerebral CT scans were also obtained from the hospital data base. These were from patients presenting for CT scanning for various indications; however no further clinical information was available. Therefore, we selected those scans (n = 40) that had a clear pathology assuming that this was the reason for the performance of the CT scan and searched for NCC related pathologies on the CT scan images. The scans were checked by two people independently, a neuroradiologist (KS) and a neurologist (JB). In case of disagreement, the scans were discussed until a consensus was achieved. CT based diagnosis of NCC was divided into three groups: definite NCC lesions, lesions highly suggestive of, and those compatible with NCC. Definite NCC lesions were cystic lesions showing the scolex while those without a visible scolex, multiple and parenchymal brain calcifications were categorized as lesions highly suggestive of NCC [23]. Compatible cases were those with any other pathology that could be caused by NCC, such as hydrocephalus [24, 25], and those with single calcifications in brain parenchyma [26]. NCC was diagnosed as being either active for any cystic lesions or inactive for only parenchymal calcifications [27, 28].

### Diagnosis of NCC

Diagnosis of NCC was based on the Del Brutto diagnostic criteria [23]. Different diagnostic findings are stratified into absolute, major and minor criteria. The combination of these criteria results in a diagnosis of “Definitive NCC”, “Probable NCC” and “No NCC” [23]. All our participants had an epidemiological criterion, because they lived in an area endemic for *T*. *solium* taeniosis/cysticercosis and all PWE had a minor criterion, because they had a clinical manifestation suggestive of NCC. Additional to the Del Brutto criteria, we also used sero-Ag-ELISA as a major criterion as proposed by Gabriël et al. [29]. Both diagnostic classifications are presented in the result section.

### Statistical analysis

Data from the screening questionnaire and all further examinations was entered into a Microsoft Excel spreadsheet (double entry and review, according to Good Data Practice (GDP) and analyzed with R (3.1.0) (http://www.r-project.org/). The statistical test used is mentioned in the text. The significance level was set to 0.05. In the analysis of age differences, cases with unknown age are excluded from the analysis. For the diagnosis of NCC missing values for minor, major and absolute criteria are set to negative.

## Results

Due to limited funding the number of PWE selected for CT scan had to be limited to a maximum of 50 individuals. One patient refused to go to Lusaka, therefore we conducted 49 CT scans. Another limitation is the selection of people with high scores in the screening questionnaire, which might have led to a selection bias of PWE. More details about the limitations of this study are mentioned in the discussion.

### Demographic data

Of the 51 PWAE, 24 (47.1%) were females. For the five PWIE, one (20.0%) was female. In the group of 40 CT control scans, 20 (50.0%) were women. There was no significant difference in gender distribution among the three groups (Fisher’s exact test, p = 0.552).

The mean age with standard deviation of the 56 people with active epilepsy and inactive epilepsy was 32.3 ± 15.3 and 28.8 ± 20.4 years, respectively. The mean age of the patients from the CT control scans was 41.4 ± 20.9 years. There was a significant difference in age among the three groups (ANOVA, p = 0.048). A Scheffe-Post-Hoc-Test revealed a borderline difference between CT controls and PWAE (p = 0.070).

### Diagnosis of epilepsy

After history taking and neurological examination of the 56 patients, epilepsy was diagnosed and classified as shown in Table 2. The majority of PWE had clinically generalized seizures with about one third (17/56) having “Generalized seizures within specific age range” and a quarter (14/56) with “Generalized seizures outside specific age range”.

**Table 2:** Diagnosis of type of epilepsy after neurological examination.

Type of seizure	Number
Generalized seizures within specific age range	17
Generalized seizures outside specific age range	14
Generalized seizures with focal signs	11
Generalized seizures with diffuse brain damage	7
Complex partial seizures	2
Inactive epilepsy	5
**Total**	56

### Laboratory tests

Of the 56 PWE, 39.3% (22/56) had *T*. *solium* cysticercosis antibodies while 23.2% (13/56) had circulating cysticercus antigens. Fifty-four PWE provided a stool sample and 14.8% (8/54) were positive for taeniosis on copro-Ag ELISA (Table 3). The diagnostic tests results according to activity of epilepsy and the CT scan result are presented in Table 3. A list with age, gender, the clinical diagnosis of epilepsy, the descriptive diagnosis of the CT scan, number of active and inactive NCC lesions, serological results and the diagnosis of NCC for every participant is shown in the supplement material S1 Table. There were no significant differences comparing results of the laboratory tests with the result of the classification of NCC lesions in CT scanning (Fisher test; EITB: p = 0.452, Ag-ELISA: p = 0.100, copro-Ag ELISA: p = 0.542).

**Table 3:** Diagnostic test results according to activity of epilepsy and CT scan results.

	Diagnostic test			
		Serum EITB	Serum Ag-ELISA	Copro-Ag ELISA
Epilepsy activity	N	No. +ve (%)	No. +ve (%)	No. +ve (%)
Inactive	5	3 (60.0)	2 (40.0)	1 (20.0)
Active	51*	19 (37.3)	11 (21.6)	7 (14.3)
Totals	56**	22 (39.3)	13 (23.2)	8 (14.8)
NCC result on CT scan of PWAE				
No NCC lesion	29	9 (31.0)	5 (17.2)	3 (10.3)
Compatible	6***	1 (16.7)	1 (16.7)	1(20.0)
Highly suggestive	12	6 (50.0)	2 (16.7)	3 (25.0)
Definite	2	1 (50.0)	2 (100)	0 (0.0)
Total	49****	17 (34.7)	10 (20.4)	7 (14.6)

*For Copro-Ag ELISA n = 49

** For Copro-Ag ELISA total n = 54

*** For Copro-Ag ELISA n = 5

**** For Copro-Ag ELISA total n = 48

PWAE… People with active epilepsy, PWIE… People with inactive epilepsy, NCC … Neurocysticercosis, CT… Computed tomography, EITB… Enzyme linked immunoelectrotransfer blot to detect cysticercus antibodies in serum, Ag-ELISA… Detection test for cysticercus antigens in serum, Copro-Ag ELISA… Detection test for antigens in stool to diagnose taeniosis

### Diagnosis of NCC based on computed tomography results

CT scanning was performed on 49 (96.1%) of the 51 PWAE. Lesions compatible with NCC were found in 12.2% (6/49), lesions highly suggestive of NCC in 24.5% (12/49) and definite NCC lesions in 4.1% (2/49) of CT scans of PWAE. This compares to 15.0% (6/40), 2.5% (1/40) and 0.0% (0/40), respectively, in the control CT scans. The difference between the two groups was statistically significant (Fisher’s exact test, p < 0.001). A detailed description of all CT scans results can be found in supplement material (S1 Table).

Two people with active epilepsy had a calcification in the *masseter muscle* visible on CT scan, which could be interpreted as cysticercosis outside the central nervous system (CNS). However, Del Brutto defines “cysticercosis outside CNS”, which is a minor criterion, as multiple calcification in thigh or calf muscles [23]. Therefore, we did not consider these calcifications as “cysticercosis outside CNS”.

### Diagnosis of NCC according to the Del Brutto criteria

Combining CT scan and serology EITB results, 15.7% (8/51) of PWAE were diagnosed with definitive and additionally 35.3% (18/51) with probable NCC as described by Del Brutto [23] (Table 4). Of the five PWIE, three (60%) were diagnosed with probable NCC. Combining definitive and probable NCC, this resulted in an overall NCC prevalence of 51.8% (29/56) among PWE. 31.0% (9/29) of people with probable or definitive NCC had no NCC lesion on CT scan.

**Table 4:** Diagnosis of neurocysticercosis according to Del Brutto (2012) and Gabriël et al. (2012) diagnostic criteria.

	NCC diagnosis according to Del Brutto, (2012, without Antigen-ELISA in serum as major criterion)			NCC diagnosis according to Gabriël et al., (2012, with using Antigen-ELISA in serum as major criterion)		
	No NCC	Probable NCC	Definitive NCC	No NCC	Probable NCC	Definitive NCC
Group	n (%)	n (%)	n (%)	n (%)	n (%)	n (%)
PWAE*	25 (49.0)	18 (35.3)	8 (15.7)	22 (43.1)	16 (31.4)	13 (25.5)
PWIE**	2 (40.0)	3 (60.0)	0 (0.0)	2 (40.0)	1 (20.0)	2 (40)
CT Controls^#^	40 (100)	0 (0.0)	0 (0.0)	40 (100)	0 (0.0)	0 (0.0)
Total	67 (69.8)	21 (21.9)	8 (8.3)	64 (66.7)	17 (17.7)	15 (15.6)

*n = 49

** n = 5, no CT scan results available

^#^ n = 40, only CT scan results available

PWAE… People with active epilepsy, PWIE… People with inactive epilepsy, NCC … Neurocysticercosis, CT… Computed tomography

### Diagnosis of NCC according to the adapted criteria proposed by Gabriël et al.

Adding the sero-Ag-ELISA results as a major diagnostic criteria, as proposed by Gabriël et al. [29], 25.5% (13/51) and 31.4% (16/51) of PWAE were diagnosed with definitive and probable NCC, respectively (Table 4). Of the five PWIE, two (40.0%) were diagnosed with definitive NCC and another one (20.0%) with probable NCC. The overall NCC prevalence in PWE was therefore 57.1% (32/56). 34.4% (11/32) of people with probable or definitive NCC had no NCC lesions on CT scan.

### Associations of NCC with age, gender and clinical diagnosis of epilepsy

Combining probable and definitive NCC into a dichotomous variable, there were no significant differences in terms of gender and age at examination between people with and those without NCC (gender: Fisher test, Gabriël et al.: p = 0.280, Del Brutto: p = 0.788, age at examination: T-test, Gabriël et al.: p = 0.817, Del Brutto: p = 0.666). Also, the age at first seizure was not significantly different between the two groups (T-test, Gabriël et al.: p = 0.894, Del Brutto: p = 0.705). There was no significant association between the clinical diagnosis of epilepsy according to Winkler et al. [20] and NCC diagnosis (Fisher test, Del Brutto: p = 0.265, Gabriël et al.: p = 0.298). The age, gender and clinical diagnosis of epilepsy are shown in Table 5.

**Table 5:** The age at examination and at first seizure, gender and clinical diagnosis of epilepsy is shown according to the Del Brutto and Gabriël et al. diagnostic criteria.

NCC diagnosis		Age at examination	Age at first seizure		Gender		Clinical diagnosis of epilepsy					
		Mean ± SD in years	Mean ± SD in years		F	M	Generalized seizures within specific age range	Generalized seizures outside specific age range	Generalized seizures with diffuse brain damage	Generalized seizures with focal signs	Complex partial seizures	Inactive epilepsy
**NCC according to Gabriël et al., 2012**	No NCC	32.6 **±** 15.3	17.0 ± 16.3	N	13	11	4	7	4	5	2	2
				%	54.2	45.8	16.7	29.2	16.7	20.8	8.3	8.3
	Def./prob.	31.6 **±** 16.0	16.4 ± 15.9	N	12	20	13	7	3	6	0	3
				%	37.5	62.5	40.6	21.9	9.4	18.8	0.0	9.4
**NCC according to Del Brutto 2012**	No NCC	31.0 **±** 15.1	15.8 **±** 15.8	N	13	14	5	8	5	5	2	2
				%	48.1	51.9	18.5	29.6	18.5	18.5	7.4	7.4
	Def./prob.	32.9 **±** 16.3	17.4 **±** 16.2	N	12	17	12	6	2	6	0	3
				%	41.4	58.6	41.4	20.7	6.9	20.7	0.0	10.3

NCC … Neurocysticercosis, F…Female, M… Male, SD… Standard deviation; *Generalized seizures within specific age range*: primary generalized seizures that start within a specific age range (mainly between 6 and 25 years when most of the idiopathic generalized epilepsies start); *Generalized seizures outside specific age range*: primary generalized seizures that lie outside the specific age range of most of the idiopathic generalized epilepsies, but have no focal start and no clinical signs of brain damage; *Generalized seizures with diffuse brain damage*: secondary generalized seizures with no clear focal start but clinically obvious diffuse brain damage; *Generalized seizures with focal signs*: secondary generalized seizures with a focal start or clear unilateral seizures but without major brain damage; *Complex partial seizures*: focal seizures with loss of consciousness (see also [20])

## Discussion

The results of this study, indicating NCC as the single most important cause of epilepsy in the study area, add to the body of knowledge on the endemicity of *T*. *solium* in Zambia, besides the reports on the parasite in pigs [17, 18, 30] and more recently in humans [15, 16]. This will therefore contribute to the burden assessment of this important, yet neglected parasite.

The prevalence of NCC among PWE reported in this study is one of the highest reported so far in the sub-Saharan African region. At a prevalence of 57.1%, based on the inclusion of the sero-Ag-ELISA test result, as proposed by Gabriël et al. [29], it ranks higher than what has been reported in the pooled world estimate of 29% [7]. It also ranks higher than those reported elsewhere in the world. In Latin America, a recent meta-analysis of epilepsy and NCC revealed an NCC proportion of 32.3% among PWE [31] with the highest being 47% reported in a CT based study in Guatemala [32]. The estimates in Africa range from 11% in Burkina Faso [7] to 37% in the Eastern Cape Province of South Africa [33] with Tanzania reporting a prevalence of 16.5% [26].

The mean age of PWAE with NCC in this study (32.9 years) is comparable to that reported elsewhere. Blocher et al. [26] reported a mean age of 32.5 years in a study in Tanzania. Though we had a limited sample size, the lack of association between NCC and age may point towards NCC being a cause of epilepsy in both the elderly and the young. Similarly, the age at first seizure was not significantly different between PWE with and without NCC, which is in line with results from Tanzania [26]. There was also no significant difference in gender distribution in our study cohort. This was similar to findings by Blocher et al. [26] in Tanzania who also did not find a significant difference regarding gender.

A prevalence of copro-Ag-ELISA positives of 14.8% among the 54 PWE may indicate a possible autoinfection with *T*. *solium* eggs that could lead to NCC. Adult tapeworm carriers are not only a risk for cysticercosis to themselves, in light of poor personal hygiene, but also to other members of their households and the community. In Mexico, Garcia-Garcia et al. demonstrated that the presence of tapeworm carriers in households is the main risk factor attributed to human cysticercosis [34]. These carriers intermittently shed proglottids and/or substantial numbers of infective eggs in their feces, thereby exposing the people in their environment to cysticercosis for example due to the unhygienic preparation and serving of food [35]. With the prevailing low standards of sanitation and personal hygiene, infection and re-infection is therefore a high possibility, hence the increased levels of cysticercosis in people living in the area. At almost 60%, we have highlighted the importance of NCC as the most important single cause of epilepsy in our study area. This may also apply to other areas of Eastern and Southern provinces of Zambia, where factors for the maintenance of the parasite have been reported and high levels of environmental contamination were highlighted by the elevated porcine cysticercosis [18, 30] and human taeniosis/cysticercosis prevalence estimates [15, 16]. A study by Mwape et al. [16] reported a high taeniosis prevalence of 11.9% in an area in Katete district, indicating a substantial number of adult tapeworm carriers with the potential to result in marked environmental contamination with the taeniid eggs. The study also reported an average cysticercosis sero-prevalence of 13.1% (sero-Ag) and 35.4% (sero-antibody(Ab)) during the study period. Incidence rates of 6300 (sero-Ag, per 100000 persons-year) and 23600 (sero-Ab, per 100000 persons-year) were also determined. The sero-reversion rates recorded were 44% and 38.7% for sero-Ag and sero-Ab, respectively, over the whole period [16]. The study by Mwape et al. showed the dynamic nature of *T*. *solium* infections and that many of the people at risk become (re)infected due to the substantial environmental contamination, with a high number turning sero-negative within a year after infection. With increased levels of (re)infection rates, the possibility of NCC becomes very likely. These results, though from a different part of the district, may explain the high prevalence of NCC in PWAE in addition to the prevailing risk factors for *T*. *solium* transmission in the study area of the current study.

The sero-prevalence of cysticercosis antibodies of 39.3% in our population of PWE is similar compared to the 35.4% (mean) reported in a population not suffering from epilepsy in another part of the district [15]. The prevalence of anticysticercal antibodies in PWE in our study was similar to the one reported in a study in South Africa (41.7%) [33], but is higher than that reported from Tanzania (15.9%) [4]. Concerning cysticercal antigens, 23.2% in our population with epilepsy compared to 13.1% in a population without epilepsy [16].

Currently, the only published criteria for the diagnosis of neurocysticercosis, which are used in epidemiological studies, are those of Del Brutto [23]. These criteria basically combine results of neuroimaging (CT and/or Magnetic Resonance Imaging) and serological diagnostic tools (circulating cysticercal antigen in cerebrospinal fluid and specific antibody detection in serum/cerebrospinal fluid). Gabriël et al. suggested an adaptation of these criteria by using sero-Ag-ELISA as a major criterion [29]. Diagnostic criteria are important in order to compare prevalence data from different countries; however they have limitations for the diagnosis of individual patients. The aim of this paper was not to discuss the different diagnostic criteria of neurocysticercosis. However, it is important to note that 34.4% (11/32) of those with a diagnosis of “Probable NCC” or “Definitive NCC” according to Gabriël et al. [29] and 31.0% (9/29) according to Del Brutto [23] have no NCC lesion on CT. Whether these patients have cysticercosis outside the central nervous system, the serological test was false positive or the NCC lesions were missed because of lack of sensitivity of a CT scan cannot be resolved. The latter has been observed in six of seven sero-Ag-ELISA positive patients with calcified NCC lesions but without viable cysts on CT scan [36].

The majority of NCC lesions were calcifications, which may support the hypothesis that mainly calcified NCC lesions are epileptogenic [27] and seizures usually develop at a later stage of the disease. When reporting low numbers of viable cysts the probably lower sensitivity of CT scan for cystic lesions has to be considered.

Two people had calcifications outside the CNS, more specifically in the masseter muscles. However, the Del Brutto criterion “Cysticercosis outside the CNS” does not include this in its description, but rather mentions multiple ‘cigar-shaped’ calcifications in the thigh and calf muscles [23]. He states that, in endemic regions, a patient may have systemic cysticercosis and neurological manifestations due to an unrelated cause. Though this is true, we nevertheless propose that evidence on either CT scan or plain X-ray films of extra CNS cysticercal lesions in the muscles of the head/thorax be included in the minor criterion “Cysticercosis outside the CNS”.

Although the data presented in this study is indeed valuable in analyzing the importance of NCC in PWE, there are some limitations. The selection of people with the highest number of positive answers in the screening questionnaire could have resulted in a bias towards more severe cases of epilepsy, i.e. with more generalized tonic-clonic seizures compared to a typical epilepsy population. We cannot rule out that there are different etiologies in different types of severity of epilepsy. Additionally, focal seizures without secondary generalization are rarely diagnosed as epileptic seizures in resource poor countries. This might have led to an underestimation of people with focal seizures in our study population. Because focal seizures have usually an underlying cause, we assume that in people with only focal seizures NCC lesions might be even more common, but we cannot prove this hypothesis with our data.

Unfortunately, there was no clinical information about the consecutive hospital cerebral CT scans available. Therefore, some patients in the consecutive scans could have had epilepsy as well and can therefore not be considered a true control group. By selecting controls with pathologic lesions other than NCC, the related symptoms of which were probably the reason for the scan, we tried to lower the probability of including PWE in the CT controls. However, we still think, it is valuable to get more information about the prevalence of NCC in a set of CT scans, because it might help to compare the prevalence of NCC among countries at a larger scale and therefore we have included the information of all 101 consecutive CT scans in the supplement material (S1 Table). In sub-Saharan Africa many countries have very few neuroimaging facilities. Consecutive images of these centers might be used as a sentinel to evaluate the NCC prevalence in a country. In a similar study in Tanzania, Winkler et al. reported a CT based prevalence of 1% of definite NCC in controls without epilepsy [4]. In the current study, the CT scans were performed only once and without application of contrast medium. As a consequence ring enhancing lesions and resolution of lesions after treatment, which are part of the Del Brutto criteria, could not be gathered. Similarly, a fundoscopic examination to visualize subretinal cysts, an X-ray of calf or thigh muscles or histology was not available, which might have led to a slight underestimation of the prevalence of NCC among PWE. In addition, in the people with the consecutive CT scans no serological tests were available, which could have also resulted in an underestimation of NCC in this group.

In conclusion, our study results of almost 60% of PWE suffering from additional NCC should be taken seriously, despite the study limitations. This is one of the highest prevalence rates based on neuroimaging reported in sub-Saharan Africa and entails the need for the commencement of concerted efforts towards the control of *T*. *solium* in endemic areas of Zambia. The findings also highlight the importance of NCC as a differential diagnosis for patients presenting with epileptic seizures not only in rural areas but also in other areas of. The present study suggests that NCC seems the most important secondary cause of epilepsy, a cause that is preventable and even eradicable [37] and therefore much more attention should be directed to its control in *T*. *solium* taeniosis/(neuro)cysticercosis endemic countries like Zambia.

## Supporting Information

S1 Checklist STROBE checklist. (DOCX)

S1 File Screening questionnaire. (DOCX)

S1 Table Table with diagnosis of epilepsy, NCC, serology results and description of CT diagnosis of PWE and controls. (XLS)

## References

[1] Murrell KD. Epidemiology of taeniosis and cysticercosis In: Murrell KD, editor. WHO/OIE/FAO Guidelines for the surveillance, prevention and control of taeniosis/cysticercosis. Paris, France: World Health Organization for Animal Health (OIE); 2005 pp. 27–43.

[2] Winkler AS. Neurocysticercosis in sub-Saharan Africa: a review of prevalence, clinical characteristics, diagnosis and management. Pathog Glob Health. 2012;106: 261–274. 10.1179/2047773212Y.0000000047 23265550 PMC4005109

[3] Carabin H, Ndimubanzi PC, Budke CM, Nguyen H, Qian Y, et al Clinical manifestations associated with neurocysticercosis: a systematic review. PLoS Negl Trop Dis. 2011;5: e1152 10.1371/journal.pntd.0001152 21629722 PMC3101170

[4] Winkler AS, Blocher J, Auer H, Gotwald T, Matuja W, Schmutzhard E. Epilepsy and neurocysticercosis in rural Tanzania—An imaging study. Epilepsia. 2009;50: 987–993. 10.1111/j.1528-1167.2008.01867.x 19054402

[5] Garcia HH, Del Brutto OH, Nash TE, White AC Jr, Tsang VC, Gilman RH. New concepts in the diagnosis and management of neurocysticercosis (*Taenia solium*). Am J Trop Med Hyg. 2005;72: 3–9. 15728858

[6] White AC Jr. Neurocysticercosis: updates on epidemiology, pathogenesis, diagnosis, and management. Annu Rev Med. 2000;51: 187–206. 10774460 10.1146/annurev.med.51.1.187

[7] Ndimubanzi PC, Carabin H, Budke CM, Nguyen H, Qian YJ et al A systematic review of the frequency of neurocyticercosis with a focus on people with epilepsy. PLoS Negl Trop Dis. 2010;4: e870 10.1371/journal.pntd.0000870 21072231 PMC2970544

[8] Goel D, Dhanai J S, Agarwal A, Mehlotra V, Saxena V. Neurocysticercosis and its impact on crude prevalence rate of epilepsy in an Indian community. Neurol India. 2011;59: 37–40. 10.4103/0028-3886.76855 21339656

[9] Preux P, & Druet-Cabanac M. Epidemiology and aetiology of epilepsy in sub-Saharan Africa. Lancet Neurol. 2005;4: 21–31. 15620854 10.1016/S1474-4422(04)00963-9

[10] Forsgren L. Estimations of the prevalence of epilepsy in sub-Saharan Africa. Lancet Neurol. 2008;7: 21–2. 18068521 10.1016/S1474-4422(07)70295-8

[11] Forsgren L, Hauser WA, Olafsson E, Sander JW, Sillanpää M, Tomson T. Mortality of epilepsy in developed countries: a review. Epilepsia. 2005;46;Suppl 11: 18–27. 16393174 10.1111/j.1528-1167.2005.00403.x

[12] Winkler AS. Epilepsy and neurocysticercosis in sub-Saharan Africa In: Foyaca Sibat H, editor. Novel aspects on cysticercosis and neurocysticercosis. InTech 2013 pp 307–340.

[13] Baskind R & Birbeck GL. Epilepsy-associated stigma in sub-Saharan Africa: the social landscape of a disease. Epilepsy Behav. 2005;7: 68–73. 15978874 10.1016/j.yebeh.2005.04.009

[14] Dorny P, Phiri IK, Vercruysse J, Gabriël S, Willingham AL 3rd, Brandt J, et al A Bayesian approach for estimating values for prevalence and diagnostic test characteristics of porcine cysticercosis. Int J Parasitol. 2004;34: 569–576. 15064121 10.1016/j.ijpara.2003.11.014

[15] Mwape KE, Phiri IK, Praet N, Muma JB, Zulu G et al *Taenia solium* infections in a rural area of Eastern Zambia-A community based study. PLoS Negl Trop Dis. 2012;6: e1594 10.1371/journal.pntd.0001594 22479664 PMC3313923

[16] Mwape KE, Phiri IK, Praet N, Speybroeck N, Muma JB, et al The incidence of human cysticercosis in a rural community of Eastern Zambia. PLoS Negl Trop Dis. 2013;7: e2142 10.1371/journal.pntd.0002142 23556026 PMC3605208

[17] Phiri IK, Dorny P, Gabriël S, Willingham AL 3rd, Speybroeck N Vercruysse J. The prevalence of porcine cysticercosis in Eastern and Southern provinces of Zambia. Vet Parasitol. 2002;108: 31–39. 12191897 10.1016/s0304-4017(02)00165-6

[18] Sikasunge CS, Phiri IK, Phiri AM, Siziya S, Dorny P Willingham AL 3rd. Prevalence of *Taenia solium* porcine cysticercosis in the Eastern, Southern and Western provinces of Zambia. Vet J. 2008;176: 240–244. 17468023 10.1016/j.tvjl.2007.02.030

[19] Birbeck GL, Kalichi EM. Epilepsy prevalence in rural Zambia: a door-to-door survey. Trop Med Int Health. 2004;9: 92–95. 14728612 10.1046/j.1365-3156.2003.01149.x

[20] Winkler AS, Schaffert M, Schmutzhard E. Epilepsy in resource poor countries—suggestion of an adjusted classification. Epilepsia. 2007;48: 1029–1030. 17509005 10.1111/j.1528-1167.2007.01009_1.x

[21] Dorny P, Brandt J, Geerts S. Immunodiagnostic approaches for detecting *Taenia solium* . Trends Parasitol. 2004;20: 259–260; author reply 260–251. 15147673 10.1016/j.pt.2004.04.001

[22] Allan JC, Avila G, Garcia Noval J, Flisser A, Craig PS. Immunodiagnosis of taeniasis by coproantigen detection. Parasitology. 1990;101 Pt 3: 473–477. 2092303 10.1017/s0031182000060686

[23] Del Brutto OH. Diagnostic criteria for neurocysticercosis, revisited. Pathog Glob Health. 2012;106: 299–304. 10.1179/2047773212Y.0000000025 23265554 PMC4005113

[24] Del Brutto OH, Rajshekhar V, White AC, Tsang VC, Nash TE, et al Proposed diagnostic criteria for neurocysticercosis. Neurology. 2001;57: 177–183. 11480424 10.1212/wnl.57.2.177 PMC2912527

[25] Del Brutto OH. Neurocysticercosis. Semin Neurol. 2005;25: 243–251. 16170737 10.1055/s-2005-917661

[26] Blocher J, Schmutzhard E, Wilkins PP, Gupton PN, Schaffert M, et al A cross-Sectional study of people with epilepsy and neurocysticercosis in Tanzania: clinical characteristics and diagnostic approaches. PLoS Negl Trop Dis. 2011;5: e1185 10.1371/journal.pntd.0001185 21666796 PMC3110162

[27] Nash TE, Del Brutto H, Butman JA, Corona T, Delgado-Escueta A, et al Calcific neurocysticercosis and epileptogenesis. Neurology. 2004;62: 1934–1938. 15184592 10.1212/01.wnl.0000129481.12067.06 PMC2912520

[28] Nash TE, Singh G, White AC, Rajshekhar V, Loeb JA, et al Treatment of neurocysticercosis: current status and future research needs. Neurology. 2006;67: 1120–1127. 17030744 10.1212/01.wnl.0000238514.51747.3a PMC2923067

[29] Gabriël S, Blocher J, Dorny P, Abatih EN, Schmutzhard E, et al Added Value of Antigen ELISA in the Diagnosis of Neurocysticercosis in Resource Poor Settings. PLoS Negl Trop Dis. 2012;6: e1851 10.1371/journal.pntd.0001851 23094118 PMC3475663

[30] Sikasunge CS, Phiri IK, Phiri AM, Dorny P, Siziya S, et al Risk factors associated with porcine cysticercosis in selected districts of Eastern and Southern provinces of Zambia. Vet Parasitol. 2007;143: 59–66. 16956727 10.1016/j.vetpar.2006.07.023

[31] Bruno E, Bartoloni A, Zammarchi L, Strohmeyer M, Bartalesi F et al COHEMI Project Study Group. Epilepsy and neurocysticercosis in Latin America: a systematic review and meta-analysis. PLoS Negl Trop Dis. 2013;7: e2480 10.1371/journal.pntd.0002480 24205415 PMC3814340

[32] Garcia-Noval J, Allan JC, Fletes C, Moreno E, Demata F et al Epidemiology of Taenia solium taeniasis and cysticercosis in two rural Guatemalan communities. Am J Trop Med Hyg. 1996;55: 282–289. 8842116 10.4269/ajtmh.1996.55.282

[33] Foyaca-Sibat H, Cowan LD, Carabin H, Targonska I, Anwary MA, et al Accuracy of Serological Testing for the Diagnosis of Prevalent Neurocysticercosis in Outpatients with Epilepsy, Eastern Cape Province, South Africa. PLoS Negl Trop Dis. 2009;3: e562 10.1371/journal.pntd.0000562 19997629 PMC2780704

[34] Garcia-Garcia ML, Torres M, Correa D, Flisser A, Sosa-Lechuga A, et al Prevalence and risk of cysticercosis and taeniasis in an urban population of soldiers and their relatives. Am J Trop Med Hyg. 1999;61: 386–389. 10497976 10.4269/ajtmh.1999.61.386

[35] Schantz PM, Moore AC, Munoz JL, Hartman BJ, Schaefer JA, Aron AM, et al Neurocysticercosis in an Orthodox Jewish community in New York City. N Engl J Med. 1992;327: 692–695. 1495521 10.1056/NEJM199209033271004

[36] Zea-Vera A, Cordova EG, Rodriguez S, Gonzales I, Pretell EJ, Castillo Y, et al Parasite antigen in serum predicts the presence of viable brain parasites in patients with apparently calcified cysticercosis only. Clin Infect Dis. 2013;57: e154–159. 10.1093/cid/cit422 23788241 PMC3765011

[37] Centres For Disease Control And Prevention. Recommendations for the International Task Force for Disease Eradication. MMWR Recomm Rep. 1993;42: 28–38. 8145708

