# Supplementary material for: Prevalence of Neurocysticercosis in People with Epilepsy in the Eastern Province of Zambia
Source: PLoS Negl Trop Dis. 2015 Aug 18;9(8):e0003972. doi: 10.1371/journal.pntd.0003972 (PMC4540454; doi:10.1371/journal.pntd.0003972)
Supplement: S1 File — (DOCX) [file pntd.0003972.s002.docx]

**Annex I: Screening questionnaire**

| **N°** | **Question** | **Answer Yes or NO** |
| --- | --- | --- |
| **SECTION ONE: NCC screening questions for epileptic seizures**  **(To be asked to each household member. For children ask their guardians.)** | | |
|  | Have you ever lost consciousness or fallen due to lost consciousness? |  |
|  | Have you ever been told that while you were unconscious your arms and legs shake or stretch out? |  |
|  | Have you ever had attacks in which you fall and bite your tongue or lost control of your bladder or bowels? |  |
|  | Have you ever had uncontrollable attacks of shaking or trembling in one arm or one leg or in the face without losing consciousness? |  |
|  | Have you ever had attacks in which you lose contact with the surroundings without losing consciousness? |  |
|  | Have you ever had attacks of losing awareness that was associated with a feeling of vagueness, unreality or dreaminess or experience of abnormal smells, sounds, or vision without losing or before loss of consciousness? |  |
|  | Have you ever been told that you had episodes of strange behavior without remembering it? |  |
|  | Have you ever been told that you have or had epilepsy or epileptic fits? |  |
| **SECTION TWO: for individuals older than 1 months and younger than 7 years at the time of the seizures (for febrile seizures; there has to be fever with every!! attack for seizures during the indicated age span!! a positive answer was given to any of the question 1-8.)** | | |
|  | Did this/these attacks occur in a child (7 years or younger) ‘only’ during an illness with fever? |  |
